# Genetic Components Derived Parameters and Heterosis in Okra under Saudi Arabia Conditions

**DOI:** 10.1155/2024/6432560

**Published:** 2024-01-23

**Authors:** Mohamed F. M. Abdelkader, Mohamed H. Mahmoud, Mohamed Z. Diyasty, Noha A. Sukar, Maged I. Farag, Nesma N. I. Mohamed, Yasser A. M. A. Salama, Mohamed A. Abdein

**Affiliations:** ^1^Department of Plant Production, College of Food and Agriculture, King Saud University, Riyadh 12372, Saudi Arabia; ^2^Department of Biochemistry, College of Science, King Saud University, Riyadh 12372, Saudi Arabia; ^3^Genetic Department, Faculty of Agriculture, Mansoura University, El-Mansoura 35516, Egypt; ^4^Agricultural Botany (Genetics) Department, Faculty of Agriculture (Girls), Al-Azhar University, Cairo, Egypt; ^5^Department of Drug Discovery Sciences, Kobe University Graduate School of Science, Technology and Innovation, Kobe, Japan; ^6^Department of Agricultural Biochemistry, Faculty of Agriculture, Ain Shams University, Cairo, Egypt; ^7^Plant Adaptation Unit-Genetic Resource Department, Desert Research Centre, Cairo, Egypt; ^8^Seeds Development Department, El-Nada Misr Scientific Research and Development Projects, Turrell, Mansoura 35511, Egypt

## Abstract

Four parental genotypes of okra were crossed in complete diallel design to study the direction and extent of relative heterosis and heterobeltiosis for yield and its associated traits for utilization of existing genetic diversity to develop heterotic *F*_1_ hybrids in okra. The additive genetic component (*D*) was significant in all studied traits except average pod weight. Nonadditive (*H*_1_ and *H*_2_) components were found to be significant in all studied traits. However, the values of the dominant effect (*H*_1_) were smaller than the *D* components for no. of nodes/plant, no. of pods/plant, weight of medium pods, weight of large pods, and total fresh pod yield. The maximum significant MP heterosis in the desirable direction (149.9%) was recorded for the weight of large pods/plot. The maximum significant heterobeltiosis in the desirable direction (120.1%) was recorded for the weight of small pods/plot followed by total fresh pod yield (107.4%), the weight of large pods/plot (104.9%), weight of medium pods/plot (92.1%), average pod weight (51.8%), number of pods/plant (38.4%), and plant height (34.3%). It could be concluded that plant height, average pod weight, and the number of branches could be considered for the development of elite hybrids (heterosis breeding) or inbred lines (pure line selection) in succeeding generations. Therefore, these parameters can be considered for selecting genotypes to improve the pod yield of okra. The superior crosses identified through heterosis analysis were Egyptian Balady × Line 4.1.18 (30.8 ton/ha), Line 4.1.18 × Egyptian Balady (29.8 ton/ha), Dwarf Green Long Pod × Line 4.1.18 (28.3 ton/ha), and Egyptian Balady × Dwarf Green Long Pod (27.6 ton/ha) as these crosses had high performance as well as significant and higher estimates of heterobeltiosis for fruit yield per plant and yield attributing other characters.

## 1. Introduction

Okra (*Abelmoschus esculentus* (L.)) belonging to the Malvaceae is a warm-season crop that is a traditional vegetable crop commercially cultivated in tropical and subtropical regions of Asia and Africa [1, 2]. Higher yields are obtained with hot weather (temperatures above 26°C), especially in regions with warm nights (>20°C) [3]. It is an annual vegetable crop that is grown for its pods consumed as vegetable [4]. According to [5], the immature fruits (pods) of okra, which are eaten as vegetables, can be used in salads, soups, and stews and fresh or dried, fried, or boiled. Notwithstanding yield, natural product quality assumes a significant part in okra efficiency and attractiveness. Except for the characteristic pod length, which is indicated by the United States Department of Agriculture [6], the criteria for defining fruit quality are not entirely clear. Fresh market okra is usually graded into three sizes, i.e., fancy: pods up to 9 cm long, choice: pods 9 cm to 11 cm long, and jumbo: pods over 11 cm but still tender [7]. These sizes are preferred in the commercial and industrial sectors, although smaller fruits are generally accepted.

A lot of variability in yield, number of days to maturity, number of pods, and plant height are showed in local varieties, and the production does not meet the demand of the population. Therefore, it is of great value to suggest better cultivars than those presently grown. Little breeding has been done on okra in Egypt or Saudi Arabia, although improved crop cultivar is one of the prerequisites for high yield. One of the tools in overcoming yield barrier and increasing productivity is heterosis breeding. Several researches have reported occurrence of high heterosis for yield and its different components [8–11]. The presence of adequate hybrid vigour is an important precondition for successful hybrid varieties production. The primary selection of involved parents in any effective hybridization programme depends upon the nature and magnitude of relative heterosis and heterobeltiosis. Heterosis exploitation is firstly dependent on the screening and selection of available germplasm that could be produced by better combinations of important traits [12]. Heterosis breeding is based on the identification of the parents and their cross combinations producing the highest level of transgressive segregates [13–17]. The choice of the best parental matings is crucial for the development of superior hybrids. The heterosis magnitude provides a guide for the choice of desirable parents for developing superior *F*_1_ hybrids. It also helps in choosing adequate crosses for commercial exploitation as well as in breeding programme. Therefore, the present work aims to study the direction and extent of relative heterosis and heterobeltiosis for yield and its related traits in 4 × 4 complete diallel crosses to use of existing genetic diversity for develop heterotic *F*_1_ hybrids in okra.

## 2. Materials and Methods

**Figure 1:**
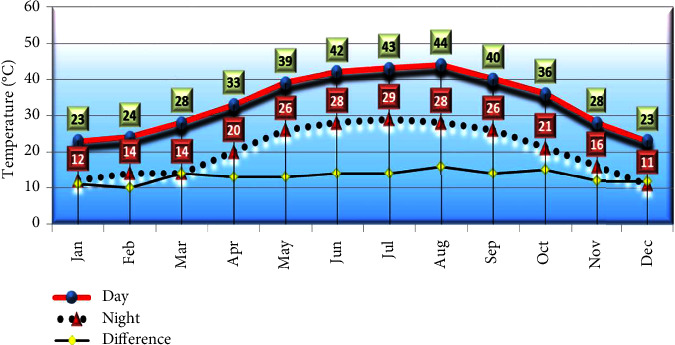
Day and night temperatures and the difference between them prevailing at Al-Kharj Governorate throughout the year (average 2019-2020).

The material used in this study consisted of four diverse genotypes of okra (*Abelmoschus esculentus* (L.) Moench), namely, *P*_1_ (Egyptian Balady cv), *P*_2_ (Line1.64.18), *P*_3_ (variety of Dwarf Green Long Pod produced by Kitazawa, Seed Company, USA), and *P*_4_ ( Line4.1.18). Both *P*_1_ and *P*_3_ were purchased locally while *P*_2_ and *P*_4_ were selected by the 1^st^ author. This experiment was conducted in Al-Kharj Governorate, Saudi Arabia, during the three summer seasons of 2018, 2019, and 2020. Five typical plants from each of the parental genotypes were selected and used to produce a 4 × 4 diallel cross including reciprocals during the summer season of 2018 in a private Farm, Al-Kharj, Saudi Arabia. The derived 12 *F*_1_ hybrids (6 single straight and 6 single reciprocal crosses) and four parents were sown on February 25^th^, 2019, in a 4-replicate randomized complete block design to assess the genotypes performance as well as recrossing and producing *F*_1_ seeds to reevaluate next season. In February 11^th^, 2020, all entries (12 *F*_1_ crosses and 4 parents) were grown in the same way as the previous season. All agronomical practices especially the irrigation and fertilization were followed to keep the crop in good condition. Selection of parents for present investigation was based on better adaptation and desirable agronomical characters. The individual plot was of 3 m length × 3 rows (30 plants/plot). Distance between rows was 60 cm and within row was 30 cm. Observation was made in parents and *F*_1_ hybrids in each replication for 14 characters, viz., plant height (PH, cm), no. of branches (NB), no. nodes/plant (NN), no. of days to flowering (Flow), pods length (PL, cm), pods diameter (PD, cm), early maturity (EM, days), number of seeds/pods (NS), number of pods/plant (NP), average pod weight (APW, g), weight of small pods/plot (WSP), weight of medium pods/plot (WMP), weight of large pods/plot (WLP), and total fresh pods (FPY, ton/ha). However, mean fresh weight (g) of okra fruits was determined by weighing fruits individually in a digital analytical balance (±0.001 g). Reported values correspond to the average of at least 50 fruits per category and per genotypes. Data were recorded during the two seasons of 2019 and 2020 and then the combined data over the two seasons were calculated and statistically analyzed. The local meteorological data in the experimental region during the studied season as an average for the 2019 and 2020 evaluation years are shown in Figure 1.

### 2.1. Statistical Analysis

Genetic components of variation from the *F*_1_ were obtained as illustrated by Hayman [18, 19]. The covariance matrix of Hayman [19] was used to provide estimates of the standard error for the genetic parameters *D*, *H*_1_, *H*_2_, and *F*. These parameters provided the estimation of the following ratios: (*H*_1_/*D*)^1/2^: measure the average degree of dominance over all loci; (*H*_2_/4*H*_1_): measure the mean value of the product *U* and *V* which are the frequencies of positive (*u*) and negative (*v*) alleles in the parents. It has a maximum value of 0.25 when *p* = *q* = 1/2. However, broad and narrow sense heritabilities were estimated according to the diallel analysis system.

### 2.2. Types of Heterosis

Relative heterosis, heterobeltiosis, and true heterosis were determined as percent increase (+) or decrease (−) of *F*_1_ over midparent (MP) and high parent (BP) in each cross using the formulae (*F*_1_ − MP/MP × 100) and (*F*_1_ − BP/BP × 100), respectively [20]. The statistical significance of heterosis and heterobeltiosis was assessed by *t*-test [21]. Heritability was based on Stanfield [22] 0 ≤ *x* ≤ 0.2 = low, 0.2 ≤ *x* ≤ 0.5 = medium, and *x* > 0.50 = high. Phenotypic (PCV %) and genotypic (GCV %) coefficients of variability were calculated according to [23]. Genetic advance (GA) was calculated with the method suggested by Johnson et al. [24] as follows: GA = *K* × *δ*^2^*g*/√*δ*^2^*p*, where *K* = 1.76, constant (on the basis of intensity of the selection 10%). Genetic advance as percent of mean (expected genetic advance) GAM % = (GA/$\overset{¯}{X}$) × 100. GAM% based on [21]: 0–7% = low, 7–14% = medium, and >14.1 = high.

## 3. Results

### 3.1. Mean Performance of the *F*_1_ Hybrids and Their Parents

High significant differences among the parental genotypes and the crosses were generally detected for all studied traits. As shown in Table 1, the mean of four parents values for plant height is 151.4 cm with a range from 133.6 (*P*_3_) to 166 cm (*P*_4_). Their 12 *F*_1_ hybrids (6 straight and 6 reciprocal crosses) ranged from 142.4 cm (*P*_2_ × *P*_4_) to 195.2 cm (*P*_4_ × *P*_3_) with a mean of 173.2 cm. Plant stems were not shorter in any of the crosses than the shortest-stem parent (over all parents). On the other hand, plants of cross *P*_2_ × *P*_4_ developed stem significantly shorter than respective shorter-stem parent. Five out of 6 crosses of one direction and all the six reciprocals had plant height significantly above the midparent value corresponding to each of them (values of average heterosis in Table 2 express this results). For number of branches per plant, the obtained data showed that the parental genotypes *P*_1_ and *P*_2_ recorded the highest number (3.9 branch/plant for each), while the lowest number of branches per plant (3.2) was obtained by the genotype *P*_3_. Among the all studied crosses, “*P*_3_ × *P*_4_,” “*P*_1_ × *P*_2_,” “*P*_2_ × *P*_1_,” and “*P*_3_ × *P*_1_” showed the highest number of branches (4.3, 4.2, 4.2, and 3.9, respectively), while the lowest number (2.9) was observed in the cross *P*_1_ × *P*_4_. The mean of four parents values for number of nodes/plant is 4.45 (Table 1) with a range of individual value from 3.4 (*P*_4_) to 5.2 (*P*_1_). The corresponding set of *F*_1_ straight hybrids had a mean value of 3.7 and ranged between 3 and 4.6 nodes. The *F*_1_ reciprocal crosses ranged from 3.4 to 5.3 with a mean of 4.1 nodes. The parental values for flowering date (Table 1) ranged from 53.9 to 57.9 days with the mean of 55.7 days. Their 6 *F*_1_ straight hybrids ranged from 53.8 to 59.8 days with a mean of 57.1 days. The mean value of flowering date of 6 reciprocal crosses is 56.2 days with a range from 54.1 to 59.8 days. The ranking of parental genotypes is as follows: *P*_2_ (earliest) and *P*_3_ (no significant differences between them) and both *P*_4_ and *P*_1_ (latest). In general, it could be noticed that *P*_2_ and the *F*_1_ straight hybrid *P*_1_ × *P*_3_ and the reciprocal hybrid *P*_2_ × *P*_1_ behaved as the earliest genotypes. The parental performance for pod length (Table 1) ranged from 5.6 cm to 6.6 cm with a mean of 6.2 cm. The genotype *P*_4_ was the best parent; meanwhile, the genotype *P*_1_ displayed the poorest value. The corresponding set of *F*_1_ straight hybrids had a mean value of 5.1 cm and ranged from 4.2 cm to 6.2 cm. The *F*_1_ reciprocal crosses ranged from 4.4 cm to 6.1 cm with a mean of 5.4 cm. Regarding pod diameter (cm), the mean parental value is 1.44 cm with a range of individual value from 1.3 cm to 1.6 cm. The corresponding set of *F*_1_ straight hybrids had a mean value of 1.33 cm and ranged from 1.13 cm to 1.44 cm. The *F*_1_ reciprocal crosses ranged from 1.12 cm to 1.40 cm with a mean of 1.28 cm (Table 1). The parental values for early maturity date (Table 1) ranged from 57.7 to 61.2 days with the mean of 59.5 days. Their 6 *F*_1_ straight hybrids ranged from 57.8 to 62 days with a mean of 60.2 days. The mean value of early maturity date of 6 reciprocal crosses is 59.7 days with a range from 58.1 to 62.6 days. The ranking of parental genotypes is as follows: *P*_2_ (earliest) and *P*_3_ (no significant differences between them) and both *P*_4_ and *P*_1_ (latest). In general, it could be noticed that *P*_2_, the *F*_1_ straight hybrid *P*_1_ × *P*_3_, and the reciprocal hybrids *P*_3_ × *P*_2_ and *P*_2_ × *P*_1_ behaved as the earliest genotypes for maturity. The mean of the parents for number of seeds per pod is 80.9 seeds with a range from 66 to 92 seeds. The corresponding set of *F*_1_ straight hybrids had a mean value of 92.51 seeds and ranged from 69.2 to 106.6 seeds. The *F*_1_ reciprocal crosses ranged from 66.3 to 97.4 seeds with a mean of 85.92 seeds.

Regarding number of pods per plant, a great variation among the studied entries (Table 1) was detected. The parental values for number of pods/plant ranged from 148.7 pods (*P*_1_) to 203 pods (*P*_4_) with the mean of 177.8 pods. Their 6 *F*_1_ straight crosses ranged from 194.9 pods (*P*_2_ × *P*_4_) to 258 pods (*P*_3_ × *P*_4_) with a mean of 232.3 pods. The mean value of this trait for 6 reciprocal crosses is 210.2 pods with a range from 179.3 pods to 239 pods. The mean performance of average pod weight for the 4 parental genotypes and their *F*_1_ hybrids (including reciprocals) is presented in Table 1.

The parental values ranged from 4 g (*P*_1_) to 5.5 g (*P*_4_) with the mean of 4.6 g. Their 6 *F*_1_ straight hybrids ranged from 4.1 g (*P*_2_ × *P*_4_) to 7.4 g (*P*_1_ × *P*_4_) with a mean of 5.9 g. The mean value of pod weight of 6 reciprocal crosses is 6.6 g with a range from 5.3 g (*P*_4_ × *P*_2_) to 7.5 g (*P*_4_ × *P*_1_). The parental mean values for weight of small pods (kg/plot) are given in Table 1. The mean of four parents' values is 7.28 kg with a range from 5.675 kg (*P*_2_) to 9.748 kg (*P*_4_). Their 12 *F*_1_ hybrids (straight and reciprocals) ranged from 5.997 kg (*P*_4_ × *P*_2_) to 16.294 kg (*P*_1_ × *P*_4_), with a mean of 11.478 kg. The corresponding set of *F*_1_ straight hybrids had a mean value of 12.529 kg and ranged from 7.286 to 16.294 kg. The *F*_1_ reciprocal crosses ranged from 5.997to 14.342 with a mean of 10.249 kg. The mean performance of the parental genotypes and their *F*_1_ hybrids for medium pods weight is presented in Table 1.

The parental genotype *P*_4_ gave the heaviest pod medium weight (24.514 kg), while values of the other three genotypes ordered as 15.135 kg (*P*_1_), 19.661 kg (*P*_2_), and 20.853 kg (*P*_3_). On the other hand, the corresponding set of *F*_1_ straight hybrids had a mean value of 35.011 kg and ranged from 24.169 to 43.404 kg. The *F*_1_ reciprocal crosses ranged from 26.139 to 42.270 kg with a mean of 32.273 kg.

As for weight of large pods, the mean of four parent values is 19.441 kg with a range from 13.314 kg (*P*_1_) to 21.02 kg (*P*_3_). Their 12 *F*_1_ hybrids (straight and reciprocals) ranged from 23.454 kg (*P*_4_ × *P*_2_) to 42.901 kg (*P*_1_ × *P*_3_), with a mean of 33.161 kg. The corresponding set of *F*_1_ straight hybrids had a mean value of 34.378 kg and ranged from 24.738 to 42.901 kg. The *F*_1_ reciprocal crosses ranged from 23.454 to 41.706 kg/plot with a mean of 32.273 kg. Data of the total fresh pod yield for 4 parents and their 12 *F*_1_ hybrids (including reciprocal) are presented in Table 1. The yield for the studied parental genotypes ranged from 9.829 (*P*_1_) to 18.612 ton/ha (*P*_4_) with a mean of 13.864 ton/ha, while the average for the *F*_1_ straight hybrids ranged from 13.471 ton (*P*_2_ × *P*_4_) to 30.845 ton (*P*_1_ × *P*_4_) with a mean of 23.134 ton. On the other hand, the average for the *F*_1_ reciprocals ranged from 15.946 ton (*P*_4_ × *P*_2_) to 29.798 ton (*P*_4_ × *P*_1_) with a mean of 23.241 ton/ha.

#### 3.1.1. Genetic Variability

The pertinent of variance components in addition to genotypic (GCV) and phenotypic (PCV) coefficients of variability for pod yield traits are presented in Table 1. Data revealed that the magnitude of phenotypic (PCV) and genotypic (GCV) coefficients of variances varied from all traits. The highest GCV and PCV were observed for all studied traits, indicating the high potential for effective selection (Burton) [25]. Small differences were observed between GCV% and PCV% for most traits, indicating the importance of the genetic effects in controlling the inheritance of these traits. Also, most studied traits had high GCV/PCV percentage. Therefore, these traits might be more genotypically predominant and it would be possible to achieve further improvement in them. Generally, the GCV/PCV exhibited the highest percentage in the parents for medium pod weight, large pod weight, and total fresh pod yield (Table 1). The heritable portion of the variance supplies the basis for plant breeders to select from phenotypic performances. Broad sense heritability based on Stanfield [22] was high for each of all studied traits in parents. High heritability indicated rapidly progress through selection for these traits. These results indicated that the environmental factors had a small effect on the inheritance of such traits. Therefore, the selection, based on mean, would be successful in improving these traits. The genotypes displayed considerable genetic advance in most traits. Genetic advance based on [21] was high for each of plant height, nodes number/plant, no. of pods/plant, and no. of seeds/plant (Table 1). Generally, high heritability was obtained for all traits along with high genetic advance as percent of the mean (GAM%).

#### 3.1.2. Genetic Components and Derived Parameters

As shown in Table 3, additive genetic component (*D*) was significant in all studied traits except average pod weight. Nonadditive (*H*_1_ and *H*_2_) components were found to be significant in all studied traits. However, the values of dominant effect (*H*_1_) were smaller than *D* components for no. of nodes/plant, no. of pods/plant, weight of medium pods, weight of large pods, and total fresh pod yield. Moreover, estimates of *H*_2_, which represent the mean dominant effect of the parents, were smaller than *H*_1_ for all studied traits.

This indicates that the frequencies of positive and negative alleles at the loci governing these traits were not equally distributed among parental genotypes. This result confirms those of *H*_2_/4*H*_1_ which deviated from its theoretical value of 0.25 in all studied traits except number of pods. The value (*H*_1_/*D*)^1/2^ was more than unity for most studied traits indicating the presence of overdominance. The ratio of additive genetic portion to the phenotypic genetic variance as indicated by heritability in narrow sense (*h*^2^*n*) was high and amounted to be 77% for no. of nodes/plant, 70.8% for total fresh pods, 69.5% for no. of pods/plant, and 0.58.7% for no. of days to flowering. On the other hand, *h*^2^*n* values were moderate for weight of large pods (54.2%), weight of medium pods (53.2%), and early maturity (41.5%). However, the estimates of narrow sense heritability were low and valued 26.5% (pods diameter), 26.1% (number of seeds/pods), and 16.4% (small pods weight), hereby selection was difficult and should be delayed to later segregating generations.

#### 3.1.3. Types of Heterosis

Heterosis for flowering date (Table 4) varied from −4.3% to 7.7% when both types of heterosis are considered. Desirable negative MP heterosis was observed in only one *F*_1_ straight cross and four reciprocal crosses, while only one *F*_1_ straight exhibited desirable BP heterosis. For plant height, the extent of variation was from −14.2% to 39.4% for both types of heterosis (Table 4) with 11 and 8 crosses showing positive MP and BP heterosis, respectively, whereas 1 and 4 crosses showing negative MP and BP heterosis, respectively. However, the important direction of heterosis for this trait either in positive or negative is depending on the breeder's point of view in respect to produce short or tall types. Heterosis values for 6 *F*_1_ hybrids and its reciprocals of number of branches are shown in Table 4. A wide range of heterosis values was existed, namely, from −21.5% to 26.2% over to midparent and from −24.6% to 20.1% over to high respective parents. Desirable positive MP heterosis was observed in 3 *F*_1_ straight crosses and 3 reciprocal crosses, while 2 straight and 3 reciprocal *F*_1_ crosses showed BP desirable heterosis.

None of the crosses showed significant positive value for nodes number in any type of heterosis (Table 4). The absence of heterosis in this trait might be due to the lower magnitude of the nonadditive type of gene action. The magnitude of significant positive heterosis for number of pods/plant was up to 48.8% over MP and 38.4% over BP. Out of 12 studied crosses (straight and reciprocals), 7 over MP and 8 over BP showed significant positive heterosis for number of pods/plant. The extent of variation for average pod weight was from −24.6% to 61.2% for both types of heterosis (Table 4), with 10 crosses showing a significant positive MP and BP heterosis, respectively. Significant positive heterosis for pod length and pod diameter up to 1.6% and 3.4% over MP, respectively, was recorded. Out of all studied crosses, 2 over MP showed significant positive heterosis for each trait, whereas none of the crosses showed positive BP heterosis for both traits. The heterotic expression for number of seeds per plant varied from −17.9% to 35% for both types of heterosis, and eight crosses showed MP and BP heterosis. Heterosis for early maturity date (Table 2) varied from −9.1% to 5.5% when both types of heterosis are considered. Desirable negative MP heterosis was observed in 2 *F*_1_ straight crosses and three reciprocal crosses, while only one *F*_1_ straight cross exhibited desirable BP heterosis. The heterotic expression for weight of small, medium, and large pod traits varied with values ranging from −38.5% to 125.7% (WSP), −1.4% to 120.2% (WMP), and 1.6% to 149.9% (WLP) for both types of heterosis with 10, 12, and 11 crosses exhibiting significant positive MP and 9, 10, and 10 crosses exhibiting significant positive BP heterosis for WSP, WMP, and WLP traits, respectively. Heterosis of total fresh pod yield expressed as the percentage deviation of *F*_1_ mean performance from MP and BP is presented in Table 4. Estimates of the 2 types of heterosis varied between −27.6% and 136.3%. Ten out of the 12 crosses showed significant positive MP heterosis and 10 crosses for BP heterosis. Generally, the best cross of each trait (due to the mean *per se*) and its heterosis and other related traits are shown in Tables 2 and 5.

## 4. Discussion

The pertinent of variance components in addition to genotypic (GCV) and phenotypic (PCV) coefficients of variability for pod yield traits were calculated. The highest GCV and PCV were observed for all studied traits in the three generations, indicating the high potential for effective selection [26]. Small differences were observed between GCV% and PCV% at the three generations for most traits, indicating the importance of the genetic effects in controlling the inheritance of these traits, i.e., NB, Flow, PL, PD, EM, APW, WSP, WLP, and FPY. Also, PH, NN, Flow, PL, PD, NS, NP, and WSP had high GCV/PCV percentage in all the three generations. Broad sense heritabilities based on [27] were high for each of all studied traits in parents, *F*_1_ straight, and *F*_1_ reciprocals. High heritability indicated rapid progress through selection for these traits. These results indicated that the environmental factors had a small effect on the inheritance of such traits. Therefore, the selection, based on mean, would be successful in improving these traits. The genotypes displayed considerable genetic advance in most traits. Generally, high heritability was obtained for all traits along with high genetic advance as percent of the mean (GAM %) in most cases. Therefore, selection for these characters would be more effective because it has high heritability and genetic advance% [28]. On the basis of the results obtained in the present study, it can be concluded that the range of variability was quite considerable for most studied characters among different genotypes. These results are in agreement partially with those obtained by [29–32].

To separate out the components of genetic variance and their ratios for all studied traits, the data were further subjected to the diallel analysis proposed by [17], although the *F*_1_ data do not entirely obey the simple additivity/dominance model in some traits where the authors in [17, 32] stated that it is possible to make estimates of parameters of genetic components even in case of partial failure of the diallel assumptions. The value (*H*_1_/*D*)^1/2^ was more than unity for most studied traits indicating the presence of overdominance. The estimates of narrow sense heritability were low and valued 26.5% (pods diameter), 26.1% (no. of seeds/pods), and 16.4% (small pods weight), hereby selection was difficult and should be delayed to later segregating generations. These results are in harmony with those obtained by [26–32].

The heterotic expression for weight of small, medium, and large pod traits varied with different values for all types of heterosis. Estimates of the two types of heterosis exhibited total fresh pod yield varied between −27.6% and 136.3%. Ten out of the 12 crosses showed significant positive MP heterosis and 10 crosses for BP heterosis. Similar magnitude of heterosis was also reported by [13, 33–39].The best crosses, which were classified on the basis of heterosis parameters, showed that two out of the 12 studied crosses were derived from *P*_4_ (Line 4.1.18) as male or female parent that was classified as a good performance for plant height, no. of pods/plant, average weight of pod, total fresh pods, weight of small pods/plot, pods length, pods diameter, and weight of medium pods. Therefore, this parent (Line 4.1.18) could be used as promising progenitors for the abovementioned traits in genetic improvements by means of selection in segregating generations. The first cross (Egyptian Balady cv × Line 4.1.18) exhibited the highest mean yield, average pod weight, WSP, WMP, and WLP. It also showed highly significant desirable heterotic effects for seven important traits, viz., NS, NP, APW, WSP, WMP, WLP, and FPY. Again, the results reveal that the cross Egyptian Balady cv × Line 4.1.18 can be considered the best combination among the 12 crosses evaluated in the present work. The another best cross, namely, (Line 4.1.18 × Egyptian Balady cv) showed high mean fruit yield/plant (29.798 ton/ha) and highly significant heterotic effects for at least six important yield contributing traits. Therefore, the abovementioned cross combinations are promising for genetic improvement either for yield or some of its important components through heterosis and/or selection in the segregating generations to exploit a fixable additive gene action.

## 5. Conclusions

Parent (Line 4.1.18) could be used as promising progenitors for plant height, no. of pods/plant, average weight of pod, total fresh pods, weight of small pods/plot, pods length, pods diameter, and weight of medium pods traits in genetic improvements by means of selection in segregating generations. The two cross combinations (Egyptian Balady cv × Line 4.1.18 *F*_1*s*_ and its reciprocal *F*_1*r*_) are promising for genetic improvement either for yield or some of its important components through heterosis and/or selection in the segregating generations to exploit a fixable additive gene action.

## Figures and Tables

**Table 1:** Range of parents, *F*_1_ straight (*F*_1*s*_), and reciprocal (*F*_1*r*_) mean performance as well as the genetic parameters at parents.

Trait	Range			Genetic parameters at parents				
	Parents	*F*_1*s*_	*F*_1*r*_	Mean ± SE	PCV	GCV	GCV/PCV	GAM%
PH	133.6–166	142.4–193.4	160.2–195.2	151.44 ± 1.59	52.05	51.21	98.39	32.37
NB	3.2–3.9	2.9–4.3	3.0–4.2	3.64 ± 0.07	0.99	0.93	93.35	6
NN	3.4–5.2	3.0–4.6	3.4–5.3	4.45 ± 0.11	4.89	4.74	97.01	29.93
Flow	53.9–57.9	53.8–59.8	54.1–59.8	55.72 ± 0.38	2.07	1.94	93.74	12.52
PL	5.6–6.6	4.2–6.2	4.4–6.1	6.17 ± 0.08	1.14	1.08	95.07	6.93
PD	1.3–1.6	1.1–1.4	1.1–1.4	1.44 ± 0.03	0.39	0.36	92.07	2.33
EM	58.8–66.7	57.8–62	58.1–62.6	59.5 ± 0.64	1.64	1.29	78.76	9.29
NS	66.0–92.0	69.2–106.6	66.3–97.4	80.93 ± 1.41	50.63	49.4	97.56	31.78
NP	148.7–203	194.9–258	179.3–239	177.8 ± 10.19	195.74	170.12	86.91	85.52
APW	3.9–5.5	4.1–7.5	5.3–7.5	4.6 ± 0.11	0.37	0.25	67.33	1.97
WSP	5.7–9.7	7.3–16.3	5.9–14.3	7.28 ± 0.06	0.9	0.77	85.07	5.27
WMP	15.1–24.5	24.2–43.4	26.1–42.3	20.04 ± 0.07	1.81	1.76	96.76	11.11
WLP	13.3–23.1	24.7–42.9	23.4–41.7	19.44 ± 0.06	12.77	12.73	99.69	78.98
FPY (ton/ha)	9.8–18.6	13.5–30.8	15.9-29-8	13.9 ± 0.27	13.57	13.22	97.47	83.23

PH: plant height (cm), NB: no. of branches/plant, NN: no. nodes/plant, Flow: no. of days to flowering, PL: pods length (cm), PD: pods diameter (cm), EM: days to maturity, NS: no. of seeds/pod, NP: no. of pods/plant, APW: average pod weight (g), WSP: weight of small pods (kg/plot), WMP: weight of medium pods (kg/plot), WLP: weight of large pods (kg/plot), and FPY: fresh pods yield (ton/ha).

**Table 2:** The best cross of each trait and its heterosis and other related traits (due to the mean).

Trait	Name	Mean performance *per se* (ton/ha)	Heterosis	
			MP	BP
PH	*P*_4_ × *P*_3_	195.18	30.30^*∗∗*^	17.59^*∗∗*^

NB	*P*_3_ × *P*_4_	4.28	26.23^*∗∗*^	20.09^*∗∗*^

NN	*P*_3_ × *P*_1_	5.28	10.54^ns^	1.41^ns^

Flow	*P*_1_ × *P*_3_	53.79	−4.33^*∗∗*^	−1.43^*∗∗*^

PL	*P*1 × *P*_4_	6.18	1.64^*∗∗*^	−6.46^*∗∗*^

PD	*P*_1_ × *P*_3_	1.44	3.35^*∗∗*^	−4.85^*∗∗*^

EM	*P*_1_ × *P*_3_	57.8	−3.64^*∗∗*^	−5.49^*∗∗*^

NS	*P*_1_ × *P*_2_	106.62	35.00^*∗∗*^	15.92^*∗∗*^

NP	*P*_3_ × *P*_4_	258	32.31^*∗∗*^	27.09^*∗∗*^

APW	*P*_4_ × *P*_1_	7.5	58.37^*∗∗*^	36.25^*∗∗*^
	*P*_1_ × *P*_4_	7.44	57.20^*∗∗*^	35.24^*∗∗*^

WSP	*P*_1_ × *P*_4_	16.29	107.30^*∗∗*^	67.16^*∗∗*^
	*P*_3_ × *P*_4_	15.27	74.81^*∗∗*^	56.67^*∗∗*^

WMP	*P*_1_ × *P*_4_	43.4	118.94^*∗∗*^	77.06^*∗∗*^

WLP	*P*_1_ × *P*_3_	39.630	149.90^*∗∗*^	104.10^*∗∗*^

FPY	*P*_1_ × *P*_4_	30.845	116.90^*∗∗*^	65.66^*∗∗*^
	*P*_4_ × *P*_1_	29.798	109.50^*∗∗*^	60.04^*∗∗*^

PH: plant height (cm), NB: no. of branches/plant, NN: no. nodes/plant, Flow: no. of days to flowering, PL: pods length (cm), PD: pods diameter (cm), EM: days to maturity, NS: number of seeds/pod, NP: no. of pods/plant, APW: average pod weight (g), WSP: weight of small pods (kg/plot), WMP: weight of medium pods (kg/plot), WLP: weight of large pods (kg/plot), and FPY: fresh pods yield (ton/ha).

**Table 3:** Genetic components and derived parameters for all studied okra traits.

Trait	*D*	*H*_1_	*H*_2_	*F*	(*H*_1_/*D*)^1/2^	*H*_2_/4*H*_1_	*h* ^2^ *b*	*h* ^2^ *n*
Plant height (cm)	231.300^*∗∗*^ ± 16.67	1413.800^*∗∗*^ ± 48.45	1188^*∗∗*^ ± 44.72	434.900^*∗∗*^ ± 42.81	2.470	0.210	0.991	0.035
No. of branches/plant	0.080^*∗*^ ± 0.03	0.482^*∗∗*^ ± 0.10	0.451^*∗∗*^ ± 0.09	0.087 ± 0.09	2.439	0.234	0.845	0.082
No. nodes/plant	0.607^*∗∗*^ ± 0.05	0.583^*∗∗*^ ± 0.14	0.502^*∗∗*^ ± 0.13	−0.371^*∗*^ ± 0.12	0.980	0.215	0.952	0.770
No. of days to flowering	2.890^*∗∗*^ ± 0.43	5.895^*∗∗*^ ± 1.26	4.235^*∗∗*^ ± 1.16	0.347 ± 1.11	1.428	0.180	0.882	0.587
Pods length (cm)	0.192^*∗∗*^ ± 0.06	3.065^*∗∗*^ ± 0.16	2.499^*∗∗*^ ± 0.15	0.661^*∗∗*^ ± 0.14	3.995	0.204	0.983	0.071
Pods diameter (cm)	0.013^*∗∗*^ ± 0.004	0.066^*∗∗*^ ± 0.01	0.051^*∗∗*^ ± 0.01	0.017 ± 0.01	2.253	0.193	0.882	0.265
Early maturity (days)	−4.740^*∗∗*^ ± 0.58	−21.660^*∗∗*^ ± 1.69	−16.600^*∗∗*^ ± 1.56	−2.406 ± 1.49	2.138	0.192	0.725	0.415
Number of seeds/pod	118.500^*∗∗*^ ± 10.07	531.800^*∗∗*^ ± 29.27	512.700^*∗∗*^ ± 27.02	45.33 ± 25.87	2.118	0.241	0.986	0.261
No. of pods/plant	52.910^*∗∗*^ ± 5.03	51.770^*∗∗*^ ± 14.63	51.480^*∗∗*^ ± 13.50	−39.39^*∗*^ ± 12.93	0.989	0.249	0.888	0.695
Average pod weight (g)	0.036 ± 0.03	0.625^*∗∗*^ ± 0.08	0.504^*∗∗*^ ± 0.07	0.138 ± 0.07	4.149	0.202	0.926	0.066
Weight of small pods/plot	0.026^*∗*^ ± 0.01	0.150^*∗∗*^ ± 0.03	0.137^*∗∗*^ ± 0.03	0.0241 ± 0.02	2.421	0.228	0.930	0.164
Weight of medium pods	0.214^*∗∗*^ ± 0.01	0.092^*∗*^ ± 0.04	0.071 ± 0.03	0.129^*∗∗*^ ± 0.03	0.656	0.193	0.710	0.532
Weight of large pods	1.690^*∗∗*^ ± 0.04	1.086^*∗∗*^ ± 0.01	0.728^*∗∗*^ ± 0.11	1.572^*∗∗*^ ± 0.10	0.802	0.168	0.955	0.542
Total fresh pods	3.991^*∗∗*^ ± 0.12	1.406^*∗∗*^ ± 0.35	1.008^*∗*^ ± 0.32	2.621^*∗∗*^ ± 0.31	0.594	0.179	0.910	0.708

**Table 4:** Range of heterosis % for studied traits and number of superior crosses showing significant desirable heterosis.

Trait	Heterosis % over				No. of significantly superior crosses on the base of			
	MP		BP		MP		BP	
	*F*_1*s*_	*F*_1*r*_	*F*_1*s*_	*F*_1*r*_	*F*_1*s*_	*F*_1*r*_	*F*_1*s*_	*F*_1*r*_
PH	−8.1 to 39.4	3.5 to 35.8	−14.2 to 34.3	−3.3 to 30.9	5	6	4	4
NB	−21.5 to 26.2	−20.1 to 11.2	−24.6 to 20.1	−23.7 to 6.1	3	3	2	3
NN	−26.7 to −9.3	−25.1 to 10.5	−38 to −12	−29.3 to 1.4	—	—	—	—
Flow	−4.3 to 7.7	−3.2 to 4.5	−1.4 to 9.6	0.4 to 6.1	1	1	1	
PL	−35 to 1.6	−32.4 to 0.9	−36.1 to −6.5	−33.6 to −3.6	1	1	—	—
PD	−24.6 to 3.4	−25 to −0.24	−27.8 to −2.8	−28.2 to −7.5	2	—	—	—
EM	−3.6 to 3.9	−9.1 to 3	−1.7 to 5.5	0.7 to 4	2	3	1	—
NS	−5.7 to 35.0	−10.9 to 24.6	−14.3 to 15.9	−17.9 to 10.6	4	4	4	4
NP	3.8 to 45	−4.5 to 48.8	−4 to 33.3	−11.7 to 38.4	5	2	5	3
APW	−17.2 to 61.2	6.4 to 59.6	−24 to 51.8	−3.1 to 49.8	5	5	5	5
WSP	−5.5 to 116.2	−22.2 to 125.7	−25.3 to 110.9	−38.5 to 120.1	5	5	5	4
WMP	9.4 to 120.2	18.3 to 117.1	−1.4 to 90	6.6 to 92.1	6	6	5	5
WLP	13.9 to 149.9	8 to 147.8	7.2 to 104.1	1.6 to 104.9	6	5	5	5
FPY	−14.8 to 132.1	0.8 to 136.3	−27.6 to 97.5	−14.4 to 107.4	5	5	5	5

PH: plant height (cm), NB: no. of branches/plant, NN: no. nodes/plant, Flow: no. of days to flowering, PL: pods length (cm), PD: pods diameter (cm), EM: days to maturity, NS: no. of seeds/pod, NP: no. of pods/plant, APW: average pod weight (g), WSP: weight of small pods (kg/plot), WMP: weight of medium pods (kg/plot), WLP: weight of large pods (kg/plot), FPY: fresh pods yield (ton/ha), MP: midparent of the cross, and BP: high parent of the cross.

**Table 5:** Heterosis % for studied traits in *F*_1_ straight and reciprocals.

Cross	Item	PH	NB	NN	Flow	PL	PD	EM	NS	NP	APW	WSP	WMP	WLP	FPY
*P*_1_ × *P*_2_	MP	21.08^*∗∗*^	8.22^*∗∗*^	−9.25	0.63^*∗∗*^	−10.61^*∗∗*^	2.72^*∗∗*^	0.92^*∗∗*^	35.00^*∗∗*^	43.23^*∗∗*^	22.63^*∗∗*^	116.2^*∗∗*^	85.75^*∗∗*^	67.41^*∗∗*^	74.79^*∗∗*^
	BP	14.27^*∗∗*^	7.57^*∗∗*^	−12.04^*∗∗*^	4.34^*∗∗*^	−15.04^*∗∗*^	−2.80^*∗∗*^	3.97^*∗∗*^	15.92^*∗∗*^	33.28^*∗∗*^	15.11^*∗∗*^	110.9^*∗∗*^	64.37^*∗∗*^	32.00^*∗∗*^	53.42^*∗∗*^

*P*_1_ × *P*_3_	MP	18.64^*∗∗*^	−5.12^*∗∗*^	−9.29	−4.33^*∗∗*^	−3.86^*∗∗*^	3.35^*∗∗*^	−3.64^*∗∗*^	−3.09^*∗∗*^	44.99^*∗∗*^	61.19^*∗∗*^	88.83^*∗∗*^	120.24^*∗∗*^	149.9^*∗∗*^	132.10^*∗∗*^
	BP	8.17^*∗∗*^	−13.09^*∗∗*^	−16.78^*∗∗*^	−1.43^*∗∗*^	−10.04^*∗∗*^	−4.85^*∗∗*^	−1.72^*∗∗*^	−6.72^*∗∗*^	30.13^*∗∗*^	51.79^*∗∗*^	67.41^*∗∗*^	90.04^*∗∗*^	104.1^*∗∗*^	97.53^*∗∗*^

*P*_1_ × *P*_4_	MP	6.02^*∗∗*^	−21.47^*∗∗*^	−17.79	1.63^*∗∗*^	1.64^*∗∗*^	−14.59^*∗∗*^	−0.20^*∗∗*^	17.23^*∗∗*^	41.42^*∗∗*^	57.20^*∗∗*^	107.3^*∗∗*^	118.94^*∗∗*^	133.5^*∗∗*^	116.9^*∗∗*^
	BP	4.82^*∗∗*^	−24.55^*∗∗*^	−32.22^*∗∗*^	2.85^*∗∗*^	−6.46^*∗∗*^	−22.44^*∗∗*^	0.36^*∗∗*^	10.02^*∗∗*^	22.50^*∗∗*^	35.24^*∗∗*^	67.16^*∗∗*^	77.06^*∗∗*^	93.11^*∗∗*^	65.66^*∗∗*^

*P*_2_ × *P*_3_	MP	39.35^*∗∗*^	7.5^*∗∗*^	−15.97	3.44^*∗∗*^	−26.40^*∗∗*^	−4.76^*∗∗*^	2.76^*∗∗*^	29.55^*∗∗*^	21.72^*∗∗*^	13.56^*∗∗*^	61.16^*∗∗*^	35.76^*∗∗*^	29.01^*∗∗*^	38.24^*∗∗*^
	BP	34.33^*∗∗*^	−2.04^*∗∗*^	−20.61^*∗∗*^	4.07^*∗∗*^	−27.62^*∗∗*^	−7.49^*∗∗*^	3.78^*∗∗*^	15.01^*∗∗*^	17.05^*∗∗*^	13.17^*∗∗*^	39.78^*∗∗*^	31.88^*∗∗*^	23.26^*∗∗*^	33.38^*∗∗*^

*P*_2_ × *P*_4_	MP	−8.13^*∗∗*^	−4.32^*∗∗*^	−26.72^*∗*^	5.46^*∗∗*^	−29.47^*∗∗*^	−24.55^*∗∗*^	2.95^*∗∗*^	−5.66^*∗∗*^	3.77	−17.23^*∗∗*^	−5.512	9.42^*∗∗*^	13.92^*∗∗*^	−14.79^*∗∗*^
	BP	−14.22^*∗∗*^	−8.60^*∗∗*^	−38.02^*∗∗*^	8.02^*∗∗*^	−31.84^*∗∗*^	−27.78^*∗∗*^	5.46^*∗∗*^	−14.25^*∗∗*^	−3.99	−24.62^*∗∗*^	−25.25^*∗∗*^	−1.409	7.188	−27.63^*∗∗*^

*P*_3_ × *P*_4_	MP	5.91^*∗∗*^	26.23^*∗∗*^	−23.66^*∗*^	7.72^*∗∗*^	−34.98^*∗∗*^	−7.38^*∗∗*^	3.94^*∗∗*^	13.88^*∗∗*^	32.31^*∗∗*^	31.56^*∗∗*^	74.81^*∗∗*^	89.78^*∗∗*^	95.40^*∗∗*^	73.34^*∗∗*^
	BP	−4.42^*∗*^	20.09^*∗∗*^	−32.16^*∗∗*^	9.65^*∗∗*^	−36.13^*∗∗*^	−8.76^*∗∗*^	5.41^*∗∗*^	10.93^*∗∗*^	27.09^*∗∗*^	19.44^*∗∗*^	56.67^*∗∗*^	75.61^*∗∗*^	92.29^*∗∗*^	51.80^*∗∗*^

*P*_2_ × *P*_1_	MP	4.62^*∗∗*^	6.76^*∗∗*^	1.52	−3.21^*∗∗*^	−1.45^*∗∗*^	−6.42^*∗∗*^	−2.17^*∗∗*^	18.13^*∗∗*^	48.76^*∗∗*^	59.60^*∗∗*^	125.7^*∗∗*^	117.1^*∗∗*^	111.5^*∗∗*^	136.3^*∗∗*^
	BP	−1.26	6.13^*∗∗*^	−1.6	0.36^*∗∗*^	−6.33^*∗∗*^	−11.45^*∗∗*^	0.79^*∗∗*^	1.44^*∗∗*^	38.42^*∗∗*^	49.81^*∗∗*^	120.1^*∗∗*^	92.07^*∗∗*^	66.74^*∗∗*^	107.4^*∗∗*^

*P*_3_ × *P*_1_	MP	15.83^*∗∗*^	11.14^*∗∗*^	10.54	−0.15	−4.75^*∗∗*^	−0.24	−0.35^*∗∗*^	−10.91^*∗∗*^	14.01	47.82^*∗∗*^	21.94^*∗∗*^	58.74^*∗∗*^	60.17^*∗∗*^	67.34^*∗∗*^
	BP	5.61^*∗∗*^	1.81^*∗∗*^	1.41	2.88^*∗∗*^	−10.88^*∗∗*^	−8.15^*∗∗*^	1.64^*∗∗*^	−14.24^*∗∗*^	2.321	39.20^*∗∗*^	8.11^*∗∗*^	36.97^*∗∗*^	30.81^*∗∗*^	42.43^*∗∗*^

*P*_4_ × *P*_1_	MP	5.40^*∗∗*^	−0.04^*∗∗*^	−5.98	4.48^*∗∗*^	0.93^*∗∗*^	−15.29^*∗∗*^	2.98^*∗∗*^	12.86^*∗∗*^	35.61^*∗∗*^	58.37^*∗∗*^	82.47^*∗∗*^	113.2^*∗∗*^	147.8^*∗∗*^	109.5^*∗∗*^
	BP	4.20^*∗*^	−3.96^*∗∗*^	−22.49^*∗∗*^	5.74^*∗∗*^	−7.11^*∗∗*^	−23.08^*∗∗*^	3.56^*∗∗*^	5.92^*∗∗*^	17.46^*∗∗*^	36.25^*∗∗*^	47.13^*∗∗*^	72.43^*∗∗*^	104.9^*∗∗*^	60.04^*∗∗*^

*P*_3_ × *P*_2_	MP	35.81^*∗∗*^	−12.38^*∗∗*^	−25.14^*∗*^	−0.1	−1.94^*∗∗*^	−4.76^*∗*^	−0.28^*∗∗*^	24.56^*∗∗*^	8.245	41.1^*∗∗*^	38.12^*∗∗*^	38.66^*∗∗*^	24.09^*∗∗*^	52.77^*∗∗*^
	BP	30.91^*∗∗*^	−20.17^*∗∗*^	−29.28^*∗∗*^	0.51^*∗∗*^	−3.56^*∗∗*^	−7.49^*∗∗*^	0.71^*∗∗*^	10.58^*∗∗*^	4.096	40.64^*∗∗*^	19.80^*∗∗*^	34.70^*∗∗*^	18.56^*∗∗*^	47.40^*∗∗*^

*P*_4_ × *P*_2_	MP	3.54	−20.09^*∗∗*^	−14.45	3.62^*∗∗*^	−29^*∗∗*^	−25^*∗∗*^	1.55^*∗∗*^	−9.64^*∗∗*^	−4.524	6.436	−22.24^*∗∗*^	18.34^*∗∗*^	8.009	0.823
	BP	−3.32	−23.66^*∗∗*^	−27.65^*∗∗*^	6.13^*∗∗*^	−31.38^*∗∗*^	−28.21^*∗∗*^	4.02^*∗∗*^	−17.87^*∗∗*^	−11.66^*∗*^	−3.069	−38.48^*∗∗*^	6.629	1.627	−14.37^*∗∗*^

*P*_4_ × *P*_3_	MP	30.30^*∗∗*^	11.20^*∗∗*^	−12.35	−0.02	−32.36^*∗∗*^	−15.18^*∗∗*^	0.01 ns	3.25^*∗∗*^	11.97	44.09^*∗∗*^	19.14^*∗∗*^	35.84^*∗∗*^	69.12^*∗∗*^	60.67^*∗∗*^
	BP	17.59^*∗∗*^	5.79^*∗∗*^	−22.10^*∗∗*^	1.77^*∗∗*^	−33.55^*∗∗*^	−16.45^*∗∗*^	1.42^*∗∗*^	0.56^*∗∗*^	7.55^*∗∗*^	30.82^*∗∗*^	6.776	25.70^*∗∗*^	66.43^*∗∗*^	40.70^*∗∗*^

^*∗*^, ^*∗∗*^Significant and highly significant, respectively; PH: plant height (cm), NB: no. of branches/plant, NN: no. nodes/plant, Flow: no. of days to flowering, PL: pods length (cm), PD: pods diameter (cm), EM: days to maturity, NS: no. of seeds/pod, NP: no. of pods/plant, APW: average pod weight (g), WSP: weight of small pods (kg/plot), WMP: weight of medium pods (kg/plot), WLP: weight of large pods (kg/plot), FPY: fresh pods yield (ton/fed), MP: midparent of the cross, and BP: high parent of the cross.

## Data Availability

Relevant data applicable to this research are within the paper.
